# Resveratrol reduces the inflammatory response in adipose tissue and improves adipose insulin signaling in high-fat diet-fed mice

**DOI:** 10.7717/peerj.5173

**Published:** 2018-06-29

**Authors:** Shibin Ding, Jinjin Jiang, Zhe Wang, Guofu Zhang, Jianli Yin, Xiaoya Wang, Sui Wang, Zengli Yu

**Affiliations:** 1 School of Public Health, Xinxiang Medical University, Xinxiang, Henan, PR China; 2 Henan Collaborative Innovation Center of Molecular Diagnosis and Laboratory Medicine, Xinxiang Medical University, Xinxiang, Henan, PR China; 3 School of Public Health, Capital Medical University, Beijing, PR China

**Keywords:** Resveratrol, High-fat diet, Inflammation, Insulin resistance, Adipose tissue

## Abstract

**Background:**

Obesity-induced glucose metabolism disorder is associated with chronic, low-grade, systemic inflammation and is considered a risk factor for diabetes and metabolic syndrome. Resveratrol (RES), a natural anti-inflammatory compound, is observed to improve glucose tolerance and insulin sensitivity in obese rodents and humans. This study aimed to test the effects of RES administration on insulin signaling and the inflammatory response in visceral white adipose tissue (WAT) caused by a high-fat diet (HFD) in mice.

**Methods:**

A total of 40 wild-type C57BL/6 male mice were divided into four groups (10 in each group): the standard chow diet (STD) group was fed a STD; the HFD group was fed a HFD; and the HFD-RES/L and HFD-RES/H groups were fed a HFD plus RES (200 and 400 mg/kg/day, respectively). The L and H in RES/L and RES/H stand for low and high, respectively. Glucose tolerance, insulin sensitivity, circulating inflammatory biomarkers and lipid profile were determined. Quantitative PCR and Western blot were used to determine the expression of CC-chemokine receptor 2 (CCR2), other inflammation markers, glucose transporter 4 (GLUT4), insulin receptor substrate 1 (IRS-1) and pAkt/Akt and to assess targets of interest involving glucose metabolism and inflammation in visceral WAT.

**Results:**

HFD increased the levels of total cholesterol, triglycerides, low-density lipoprotein cholesterol and proinflammatory cytokines in serum, decreased the high-density lipoprotein cholesterol level in serum, and induced insulin resistance and WAT inflammation in mice. However, RES treatment alleviated insulin resistance, increased the expressions of pAkt, GLUT4 and IRS-1 in WAT, and decreased serum proinflammatory cytokine levels, macrophage infiltration and CCR2 expression in WAT.

**Conclusion:**

Our results indicated that WAT CCR2 may play a vital role in macrophage infiltration and the inflammatory response during the development of insulin resistance in HFD-induced obesity. These data suggested that administration of RES offers protection against abnormal glucose metabolism and inflammatory adaptations in visceral WAT in mice with HFD-induced obesity.

## Introduction

The global prevalence of obesity has increased rapidly in recent years, and the number of overweight or obese adults worldwide is expected to reach 3.3 billion by 2030 (Kelly et al., 2008). The rapid increase in obesity worldwide will lead to an increasing in related health complications, including insulin resistance, type 2 diabetes mellitus (T2DM), nonalcoholic fatty liver disease and cardiovascular disorders. Accumulating animal and human research evidence implicates obesity and related metabolic disturbances as the major risk factors for the development of insulin resistance and T2DM (Qatanani & Lazar, 2007). Obesity is a chronic, low-grade inflammation that increases various inflammatory reactions related to body fat (Pradhan, 2007). White adipose tissue (WAT), a very important endocrine organ, consists mainly of subcutaneous adipose tissue (SAT) and internal adipose tissue. Internal adipose tissue makes up approximately 20% of the total body fat mass and mainly consists of visceral adipose tissue (VAT). In obesity, inflammation can occur due to increased levels of chemokines (such as CCL2 and CXCL1) and inflammatory cytokines (such as tumor necrosis factor α (TNF-α) and interleukin-6 (IL-6)) secreted from accumulated fat in hepatocytes and adipocytes, causing insulin resistance (Kim et al., 2017; Xu et al., 2003). In obesity, macrophage infiltration in adipose tissue was observed in both rodents and humans (Fjeldborg et al., 2014; Harford et al., 2011; Michaud et al., 2012). Macrophages are important immune cells, and the activation of macrophages could release various cytokines that promote the development of insulin resistance (Park et al., 2016; Zheng et al., 2015). The above studies indicate that reducing the levels of chemokines, secreted inflammatory cytokines and macrophage infiltration in VAT may alleviate obesity-induced insulin resistance and its progression.

CC-chemokine receptor 2 (CCR2), a receptor for monocyte chemoattractant proteins (MCPs), plays a pivotal role in the entry of innate immune cells into tissue and influences systemic insulin resistance and adipose tissue inflammation associated with obesity in high-fat-fed murine models (Weisberg et al., 2006). Studies have demonstrated that CCR2 deficiency could reduce the migration of macrophages in adipose tissue (Bolus et al., 2015). Pharmacological inhibition of CCR2 can reduce inflammation in adipose tissue, alter hepatic metabolism and ameliorate multiple diabetic parameters in mouse models of T2DM and high-fat diet (HFD)-fed mice (Kim et al., 2015; Sullivan et al., 2013). Moreover, deficiency of CCR2 in macrophages alleviates inflammation of adipose tissue and the associated metabolic syndrome (abnormal glucose tolerance, insulin sensitivity profiles and hepatic steatosis) in obese mice (Kim et al., 2016). Resveratrol (RES, 3,4′,5-trihydroxystilbene) is a polyphenol mainly found in grapes, mulberries and red wine, and it provides many health benefits including cardioprotective and anti-inflammatory effects. RES inhibits monocyte CCR2 binding activity and expression in a NO-, MAPK- and PI3K-dependent manner in THP-1 monocytes (Cullen et al., 2007). Furthermore, RES metabolites showed anti-inflammatory properties via regulating chemokines in a lipopolysaccharide-activated U-937 macrophage model (Schueller, Pignitter & Somoza, 2015). Recent studies demonstrated that RES ameliorates inflammation and insulin resistance in murine models of obesity induced by a HFD (Côté et al., 2015; Pan et al., 2015; Zhao et al., 2016). However, whether RES treatment could inhibit macrophage infiltration and regulate insulin signaling in insulin-sensitive WAT in obesity is still unknown.

Considering the key role of the innate immune system in obesity-induced inflammation, the regulatory function of CCR2 on inflammation and the recruitment of macrophages to insulin-sensitive tissues in response to HFD-induced obesity, we investigated the role of CCR2 in abnormal glucose disorder and inflammation of adipose tissue induced by HFD. We also determined whether RES administration would alleviate inflammation and the effects of disordered glucose metabolism induced by HFD treatment by inhibiting CCR2 expression and enhancing insulin sensitivity in VAT and SAT in obese mice.

## Materials and Methods

### Chemicals and reagents

Resveratrol (3,4,5-trihydroxy-trans-stilbene) was obtained from Sigma-Aldrich (St. Louis, MO, USA). Total cholesterol (TC), triglycerides (TG), high-density lipoprotein cholesterol (HDL) and low-density lipoprotein cholesterol (LDL) assay kits for estimation of TG, cholesterol, HDL and LDL in serum were purchased from BIOSINO Bio-technology and Science Inc. (Beijing, China). An insulin ELISA kit was obtained from Bai Wo (Shanghai, China). TRIzol and the oligonucleotides for PCR were obtained from Invitrogen, Inc. (Carlsbad, CA, USA), and a real-time quantitative PCR kit was purchased from TAKARA Bio, Inc. (cat. RR037A and RR420A; Otsu, Shiga, Japan). Mouse anti-β-actin antibody (cat. A5441) was obtained from Sigma-Aldrich (St. Louis, MO, USA), rabbit anti-Akt (protein kinase B) (cat. 4691T), rabbit anti-phosphorylated (p)-Akt (phosphorylated at Ser^473^) (cat. 4058T), rabbit anti-insulin receptor substrate 1 (IRS-1) (cat. 2390S) and rabbit anti-glucose transporter 4 (GLUT4) (cat. 2213S) antibodies were purchased from Cell Signaling Technology (Billerica, MA, USA). Rabbit anti-CCR2 antibody (cat. DF2711) and goat anti-rabbit IgG (H+L)-HRP (cat. S0001, dilution, 1:5,000) and goat anti-mouse IgG (H+L)-HRP (cat. S0002, dilution, 1:5,000) secondary antibodies were purchased from Affinity Biosciences, Inc. (Cincinnati, OH, USA). All other chemicals were of the highest grade commercially available.

### Animals and animal care

A total of 40 6-week-old male C57BL/6 mice (19–25 g) were purchased from the Vital River Laboratory Animal Technology Co., Ltd. (Beijing, China). All animals were maintained at 21 °C on a 12 h light/12 h dark cycle and were allowed access to food and water ad libitum. The protocols and the use of animals were approved by and in accordance with the Xinxiang Medical University Animal Care and Use Committee (XXMU-2016-0007). The animals were treated humanely and with regard for alleviation of suffering.

### Experimental design

After one week of acclimation, all mice were randomly divided into four groups (*n* = 10) and treated for 18 weeks as follows: (1) the standard chow diet (STD) group was fed a STD; (2) the HFD group was fed a HFD (41.26% of calories from fat); (3) the HFD-RES/L group was fed a HFD and treated with RES (200 mg/kg/day); and (4) the HFD-RES/H group was fed a HFD and treated with RES (400 mg/kg/day). The L and H in RES/L and RES/H stand for low and high, respectively. The doses of RES treatment (200 and 400 mg/kg/day) are based on those used in previous studies (Ding et al., 2017; Lagouge et al., 2006). The HFD intervention and RES treatment were started simultaneously. RES was dissolved in 0.1 mL deionized water and administered to the treatment groups daily (9:00 a.m.) by oral gavage, and the mice in the STD group and HFD group were administered with 0.1 mL deionized water by oral gavage at the same time. The RES and deionized water were administered seven consecutive days per week for 18 weeks. The macronutrient contents of the STD and the HFD are shown in Table 1, and the ingredient composition of the HFD was as follows (w/w): standard chow, 60%; custard powder, 8%; lard, 12%; sugar, 12%; peanut powder, 6%; and milk, 1% (Ding et al., 2014). The mice were weighed each week.

**Table 1:** The energy supply for the standard chow diet (STD) and the high-fat diet (HFD).

	Fat (%)	Carbohydrates (%)	Protein (%)	Total energy (kcal/g)
STD	13.68	64.44	21.88	3.29
HFD	41.26	39.61	19.13	4.59

**Note:**

STD, standard chow diet; HFD, high-fat diet.

After 18 weeks of treatment, mice were sacrificed by intravenous pentobarbital injection (20 mg/kg). Abdominal SAT and VAT (perirenal) were collected, weighed, immediately frozen in liquid nitrogen and stored at −80 °C for future experiments. The remaining SAT and VAT were fixed in a formaldehyde solution for histology and immunohistochemistry. The SAT coefficient and VAT coefficient were calculated (SAT weight and VAT weight × 100/body weight, respectively).

### Blood glucose homeostasis and insulin resistance

After 17 weeks of treatment, the intraperitoneal glucose tolerance test (IPGTT) was conducted in the mice as previously described (Marmugi et al., 2012). Briefly, mice were fasted for 10 h, and glucose (1 mg/g body weight) was injected intraperitoneally for IPGTT. Blood samples were collected from the tail vein and blood glucose measurement was conducted with a commercial hand-held glucometer (Accu-Chek Active; Roche, Shanghai, China) at the indicated time points (0, 15, 30, 60, and 120 min). Additionally, the area under the curve (AUC), an index of glucose tolerance, was calculated. The homeostasis model assessment for insulin resistance index (HOMA-IR) was calculated using the formula HOMA-IR = (fasting serum insulin concentration) × (fasting serum glucose concentration)/22.5 (Ding et al., 2014), and the insulin sensitivity index (ISI) was calculated as follows: 1/ln (fasting serum insulin concentration × fasting serum glucose concentration) (Wang et al., 2013).

### Analysis of metabolic parameters

At the end of the treatment (18 weeks), blood was collected from mice that had been fasted for 12 h. Serum was obtained after centrifugation (1,000×*g* for 10 min at 4 °C) (Eppendorf 5810R, Hamburg, Germany). The levels of TG, TC, HDL and LDL in serum were assayed using enzymatic colorimetric assays (BIOSINO Bio-technology and Science, Inc., Beijing, China) according to the manufacturer’s instructions.

### Measurement of serum cytokines, leptin, adiponectin and insulin by ELISA

Serum insulin was determined using a commercial mouse ELISA kit (Bai Wo Bio-technology, Shanghai, China). TNF-α, MCP-1 and IL-6 levels in serum were measured by mouse ELISA kits (R&D Systems, Billerica, MA, USA) according to the manufacturer’s protocols.

### Immunohistochemistry

Subcutaneous adipose tissue and VAT were fixed overnight at room temperature in formaldehyde solution (4%), dehydrated, and embedded in paraffin. Immunohistochemistry for macrophage marker F4/80 in adipose tissue was performed as described by the manufacturer’s instructions. Sections (5 μm) were incubated overnight at 4 °C with rat anti-F4/80 antibody (cat. Ab16911, diluted 1:1,000; Abcam, Cambridge, UK) and incubated for 1 h with the appropriate secondary antibody. Image-Pro Plus version 6.0 software (Media Cybernetics, Silver Spring, MD, USA) was used to perform the analysis.

### Quantitative real-time PCR

Real-time PCR was performed using RNA extracted from SAT and VAT of the experimental mice. RNA was isolated using TRIzol reagent (cat. 15596026; Invitrogen, Carlsbad, CA, USA) according to the manufacturer’s instructions. Total RNA was then converted into cDNA using a cDNA reverse transcription kit (TAKARA Bio Inc., Otsu, Shiga, Japan). Real-time PCR was performed using the SYBR Green detection system on an ABI PRISM 7900 machine (Applied Biosystems, Foster City, CA, USA) under the same conditions: one cycle of 95 °C, 5 s; 40 cycles of 95 °C, 10 s and 57 °C, 30 s. Gene expression levels were calculated using the 2^−▵▵△△CT^ method, and β-actin was used as an endogenous control gene. The sequences are listed in Table 2.

**Table 2:** Primer sequences used for real-time PCR.

Gene	Forward primer (5′–3′)	Reverse primer (5′–3′)
*CCR2*	TCATCCACGGCATACTATCAACA	GTGGCCCCTTCATCAAGCT
*MCP-1*	CCACTCACCTGCTGCTACTCA	TGGTGACCTCTTGTAGCTCTCC
*TNF-α*	CCCAGACCCTCACACTCAGATC	GCCACTCCAGCTGCTCCTC
*IL-6*	CTGCAAGAGACTTCCATCCAGTT	AGGGAAGGCCGTGGTTGT
*F4/80*	CTTTGGCTATGGGCTTCCAGTC	GCAAGGAGGACAGAGTTTATCGTG
*GLUT4*	CCCTGTTACCTCCAGGTTG	CCTTGCCCTGTCAGGTATGT
*IRS-1*	GCCAATCTTCATCCAGTTGC	CATCGTGAAGAAGGCATAGG
*β-actin*	TTCGTTGCCGGTCCACACCC	GCTTTGCACATGCCGGAGCC

**Note:**

CCR2, CC-chemokine receptor 2; MCP-1, monocyte chemo-attractant protein 1; TNF-α, tumor necrosis factor α; IL-6, interleukin-6; GLUT4, glucose transporter 4; IRS-1, insulin receptor substrate.

### Western blot analysis

Subcutaneous adipose tissue and VAT were homogenized with RIPA protein extraction reagent (cat. P0013B; Beyotime, Jiangsu, China) on ice. Equal quantities of protein were loaded and separated on a 10% and 7.5% SDS–PAGE gels. After electrophoresis, proteins were transferred to immobilon-P polyvinylidene difluoride membranes and blocked with 5% nonfat milk. Then, the membranes were immunoblotted with different primary antibodies: CCR2 (dilution, 1:2,000), IRS-1 (diluted 1:1,000), GLUT4 (diluted 1:1,000), Akt (diluted 1:1,000), pAkt (phosphorylated at Ser^473^) (diluted 1:2,000) and β-actin (diluted 1:10,000; Sigma-Aldrich, St. Louis, MO, USA). After the membranes were washed, the immunoblots were incubated with a secondary antibody conjugated with horseradish peroxidase, visualized with an ECL detection system (Syngen, Cambridge, UK) and analyzed using ChemiDoc Quantity One software (Bio-Rad Laboratories, Milan, Italy). β-actin was used as a loading control for CCR2, and pAkt was normalized to Akt.

### Statistical analyses

The data are shown as the mean ± SD. All statistical analyses were conducted using SPSS13.0 (SPSS, Chicago, IL, USA). Repeated measures ANOVA, with time (week) and diet (STD or HFD) as repeated measures were used to analyze body weight. The Greenhouse–Geisser test was used to revise degrees of freedom when Mauchly’s test of sphericity showed *P* < 0.05. The body weight gain, SAT coefficient, VAT coefficient, serum TG, serum TC, serum HDL, serum LDL, serum MCP-1, serum TNF-α, serum IL-6, AUC, ISI, HOMA-IR, and serum glucose at different time points in the IPGTT, as well as mRNA and protein expression levels were analyzed for statistical significance by one-way ANOVA followed by post hoc analysis (Bonferroni post-test). A value of *P* < 0.05 was considered significant.

## Results

### Effects of RES treatment on body weight curve, body weight gain and SAT/VAT coefficient

**Figure 1:**
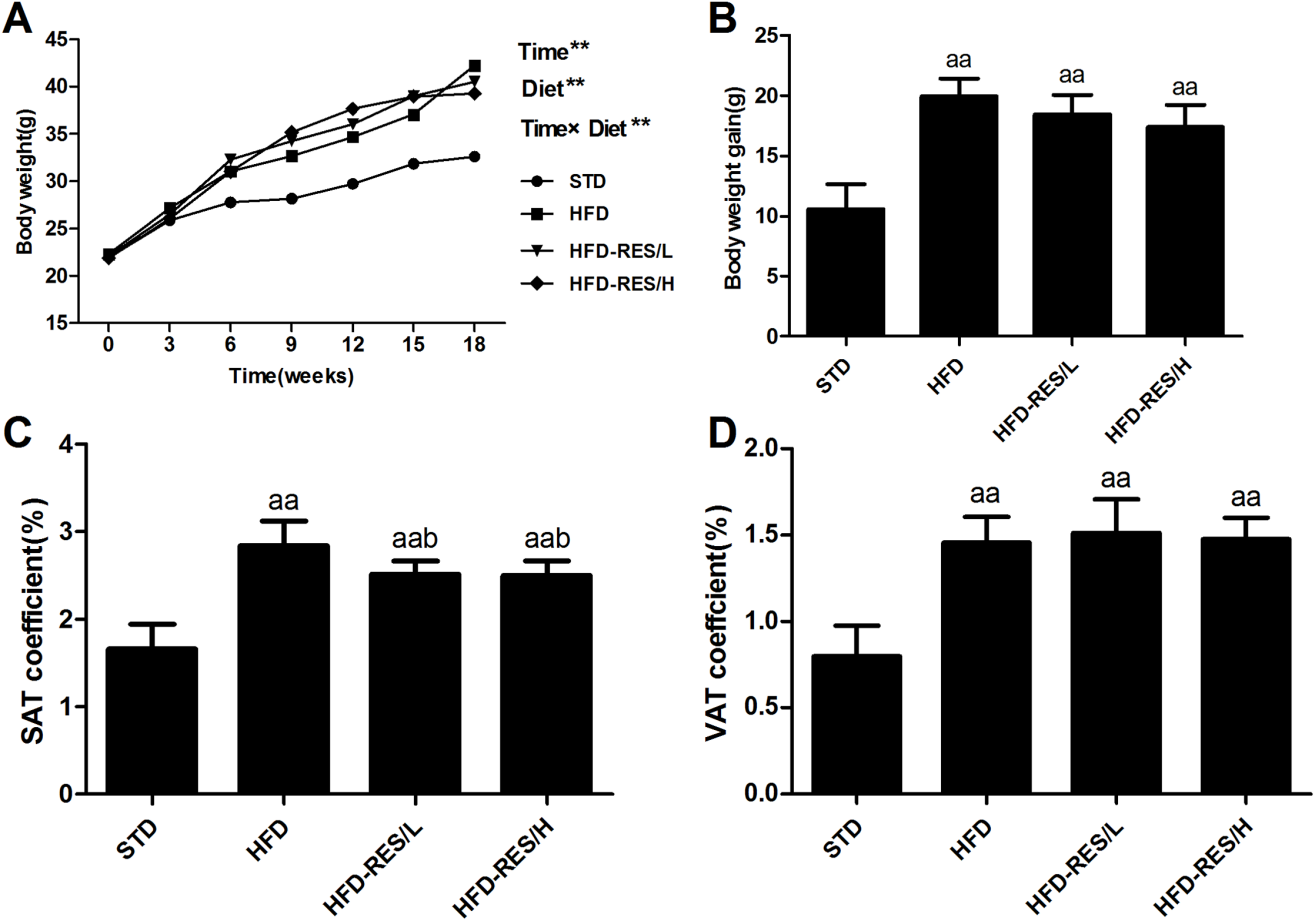
Effects of RES treatment on body weight curve, body weight gain and subcutaneous/visceral adipose coefficients of mice in response to resveratrol treatment. (A) Body weight curve; (B) Body weight gain; (C) SAT coefficient; (D) VAT coefficient. ^aa^, *P* < 0.01 vs. the STD group; ^b^, *P* < 0.05 vs. the HFD group. Time** indicates exposure duration. Diet** indicates the variety of diet. Time × diet** indicates the interaction effect between treatment duration and variety of diet. Data are expressed as the mean ± SD.

As shown in Fig. 1, after 18 weeks of treatment, the HFD-fed mice had significantly increased body weight compared to that of the STD-fed mice (*P* < 0.01), and there was no significant difference in body weight gain between the HFD group and the RES-treated groups (*P* > 0.05). The SAT coefficient and VAT coefficient in the HFD-fed groups were significantly higher than those in the STD-fed group (*P* < 0.01). RES treatment significantly decreased the SAT coefficient in the HFD-fed mice (*P* < 0.05). However, RES treatment did not affect the VAT coefficient in the HFD-fed mice (*P* > 0.05).

### Effects of RES treatment on glucose homeostasis in mice

**Figure 2:**
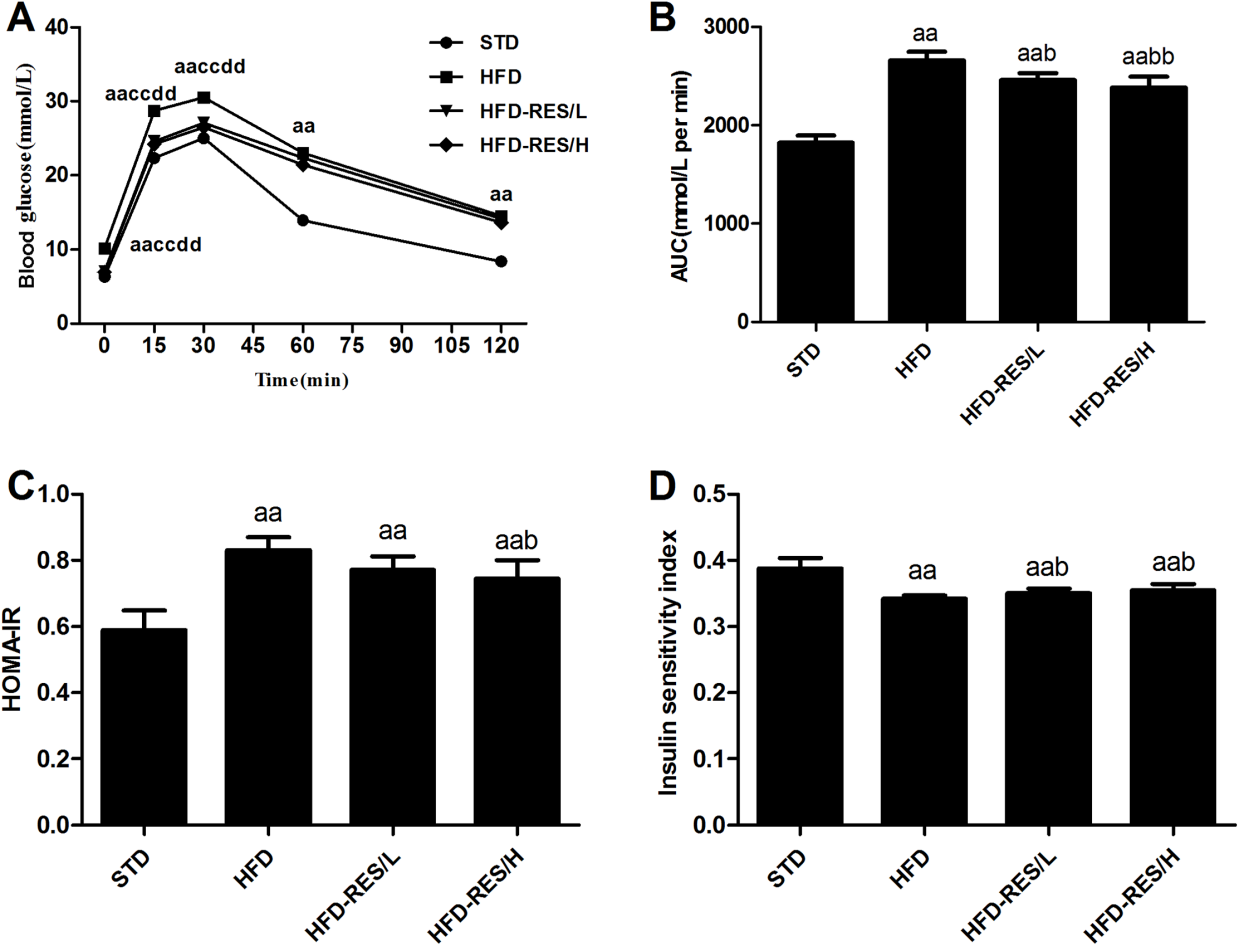
Effects of resveratrol treatment on glucose homeostasis in mice. (A) Blood glucose during IPGTT after 17-week treatment; (B) AUC of IPGTT; (C) HOMA-IR; (D) Insulin sensitivity index.

To assess the effects of RES treatment on glucose tolerance in HFD-fed mice, we measured blood glucose levels at different time points during the IPGTT after the 17-week treatment. As shown in Fig. 2, the blood glucose levels of the HFD group displayed a marked increase compared with those of the STD group at all time points and compared with those of the HFD-RES/L group and HFD-RES/H group at 0, 15 and 30 min (*P* < 0.01). The AUC was significantly increased in the HFD groups vs. that in the STD group (*P* < 0.01). Moreover, the AUC was significantly decreased in the RES-treated groups when compared to that in the HFD group (*P* < 0.01 or *P* < 0.05). Compared to those in the STD group, significantly increased HOMA-IR values (*P* < 0.01) and significantly decreased insulin sensitivity were observed in the HFD-fed groups (*P* < 0.01). Furthermore, compared to those in the HFD group, significantly decreased HOMA-IR values (*P* < 0.05) and increased ISI were observed in the HFD-RES/H group (*P* < 0.05).

### Effects of RES treatment on serum metabolic parameters

**Figure 3:**
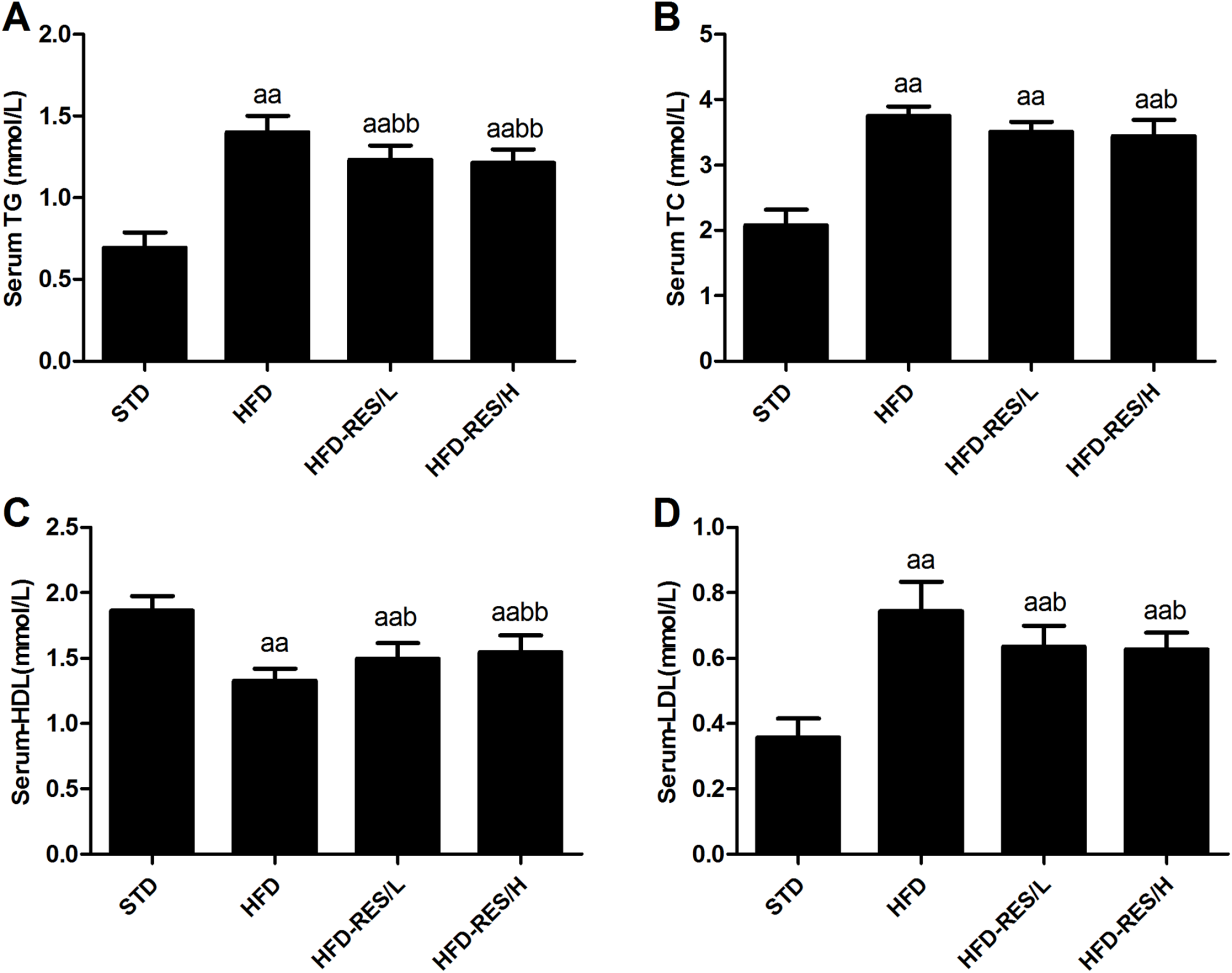
Effects of resveratrol treatment on serum metabolic parameters. (A) Serum TG; (B) Serum TC; (C) Serum HDL; (D) Serum LDL. ^aa^, *P* < 0.01 vs. the STD group; ^b^, *P* < 0.05 and ^bb^, *P* < 0.01 vs. the HFD group. The data are represented as the mean ± SD.

Figure 3 shows the serum metabolic parameters and hepatic lipid levels after HFD treatment and RES administration. The TG, TC and LDL levels in serum of the HFD-fed groups were significantly higher than those in the STD group (*P* < 0.01). Furthermore, decreased serum HDL was observed in the HFD-fed groups compared to that in the STD group (*P* < 0.01). In addition, RES (400 mg/kg/day) treatment significantly decreased the TG and TC levels in serum of the HFD-fed mice (*P* < 0.01 or *P* < 0.05). Compared to those in the HFD group, significantly increased serum HDL levels and decreased LDL levels were observed in the RES-treated groups (*P* < 0.01 or *P* < 0.05). No significant differences in TG, TC, HDL and LDL levels in the serum were observed between the HFD-RES/L group and HFD-RES/H group (*P* > 0.05).

### Effects of RES treatment on serum cytokines

**Figure 4:**
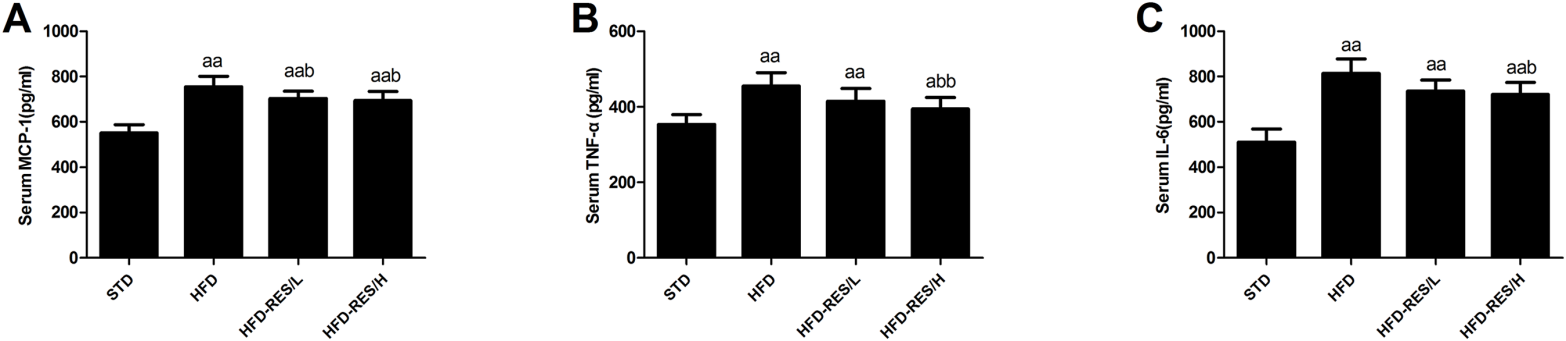
Effects of resveratrol treatment on serum cytokines. (A) MCP-1; (B) TNF-α; (C) IL-6. ^a^, *P* < 0.05 and ^aa^, *P* < 0.01 vs. the STD group; ^b^, *P* < 0.05 and ^bb^, *P* < 0.01 vs. the HFD group. The data are represented as the mean ± SD.

As shown in Fig. 4, the levels of serum MCP-1, TNF-α and IL-6 in the HFD-fed groups were markedly increased compared to those in the STD group (*P* < 0.01). RES (400 mg/kg/day) treatment decreased the serum MCP-1, TNF-α and IL-6 levels in the HFD groups (*P* < 0.05 or *P* < 0.01). However, the low dose of RES (200 mg/kg/day) treatment only decreased the serum MCP-1 level in HFD-fed mice (*P* < 0.05).

### Effects of RES treatment on mRNA and protein expression of a macrophage marker in SAT and VAT

**Figure 5:**
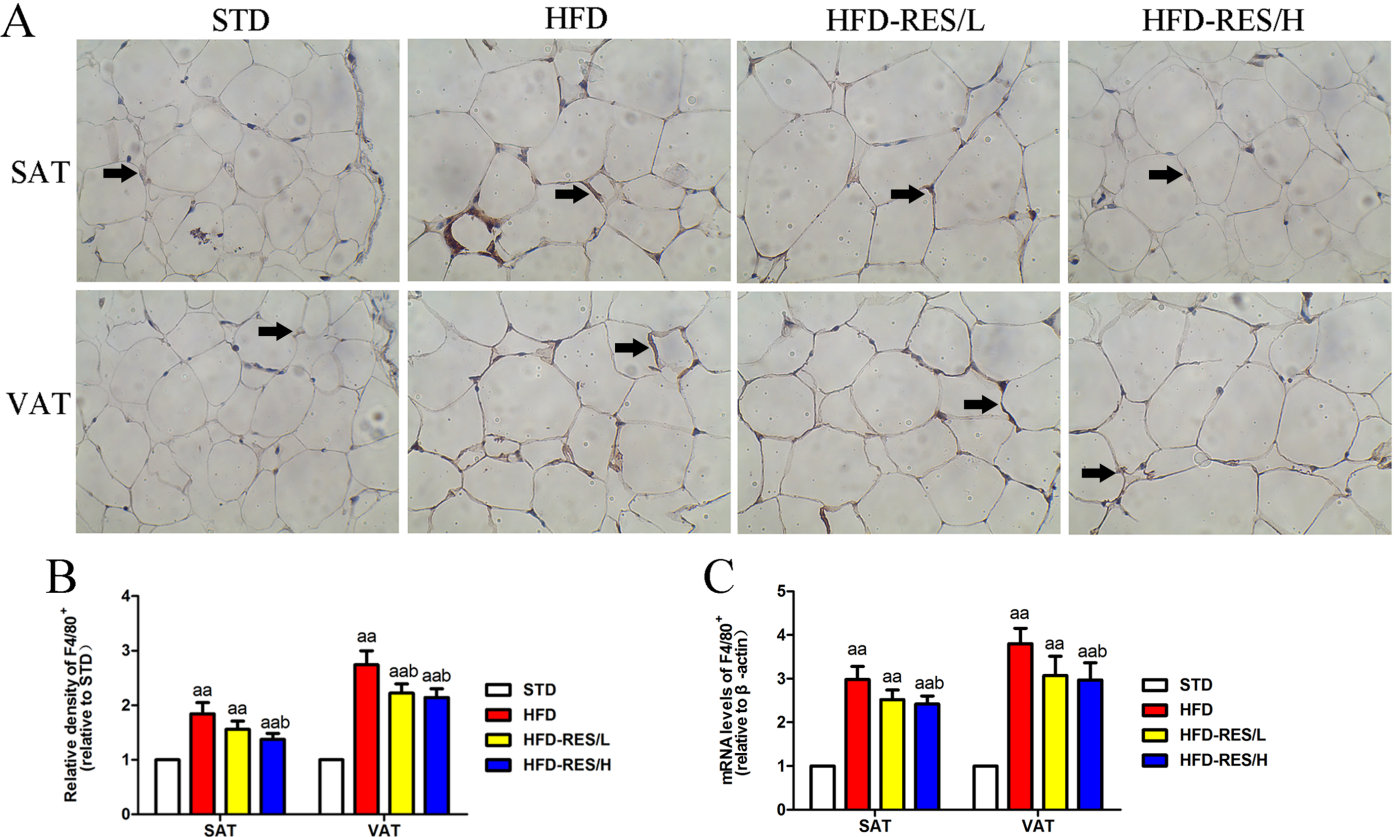
Effects of resveratrol treatment on mRNA and protein expression levels of a macrophage marker in SAT and VAT. (A) Immunohistochemistry staining of F4/80 (total macrophages) in SAT and VAT; (B) quantitative expression of F4/80 (total macrophages) is shown as relative density in SAT and VAT; (C) the mRNA expression of F4/80 in SAT and VAT. Original magnification is ×200 (scale bar = 100 μm). ^aa^, *P* < 0.01 vs. the STD group; ^b^, *P* < 0.05 vs. the HFD group. The data are represented as the mean ± SD.

We used immunohistochemistry and real-time PCR with the macrophage marker F4/80 to assess the infiltration of total macrophages in SAT and VAT (See Fig. 5). Significantly increased F4/80 mRNA and protein expression in SAT and VAT were observed in the HFD groups when compared to that in the STD group (*P* < 0.01). Compared to that in the HFD group, the mRNA and protein expression of F4/80 was significantly decreased in RES-treated mice (*P* < 0.05).

### Effects of RES treatment on mRNA expression in SAT and VAT of mice

**Figure 6:**
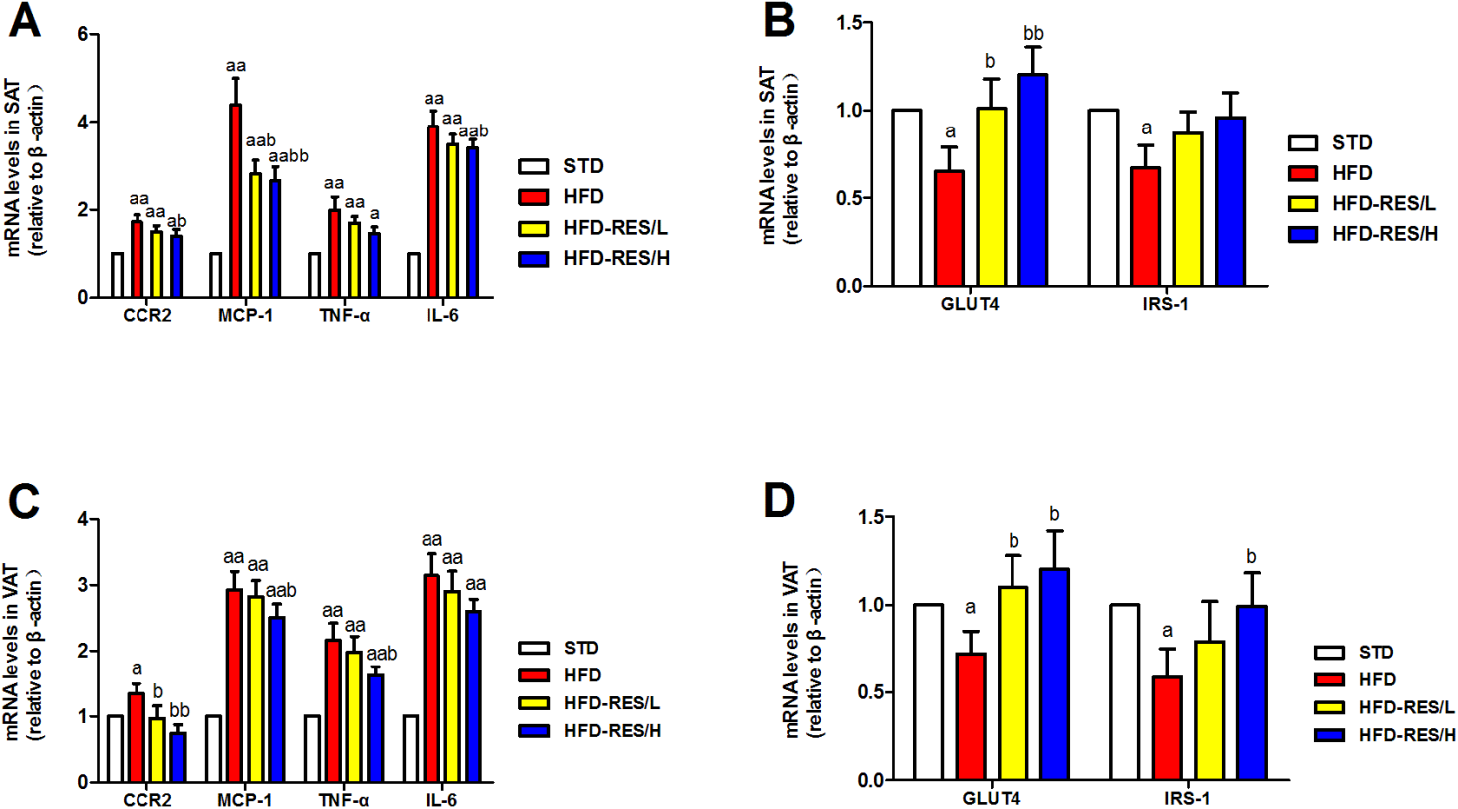
Effects of resveratrol treatment on the target mRNA expression in SAT and VAT. (A) The mRNA levels of inflammatory cytokines (*CCR2*, *MCP-1*, *TNF-α* and *IL-6*); (B) the mRNA levels of *GLUT4* and *IRS-1* in SAT; (C) the mRNA levels of inflammatory cytokines (*CCR2*, *MCP-1*, *TNF-α* and *IL-6*) in VAT; (D) the mRNA levels of *GLUT4* and *IRS-1* in VAT. ^a^, *P* < 0.05 and ^aa^, *P* < 0.01 vs. the STD group; ^b^, *P* < 0.05 and ^bb^, *P* < 0.01 vs. the HFD group. The data are represented as the mean ± SD.

As shown in Fig. 6, we determined the effect of RES administration on the mRNA expression of inflammatory cytokines (*CCR2*, *MCP-1*, *TNF-α* and *IL-6*), *GLUT4* and *IRS-1* in SAT and VAT. Levels of *CCR2*, *MCP-1*, *TNF-α* and *IL-6* in SAT and VAT were significantly increased in the HFD groups compared to those in the STD group (*P* < 0.01). RES administration significantly decreased the mRNA levels of *CCR2*, *MCP-1* and *IL-6* in SAT in the HFD mice compared to that in the HFD mice (*P* < 0.01 or *P* < 0.05). Moreover, RES administration significantly decreased the mRNA levels of *CCR2*, *MCP-1* and *TNF-α* in VAT in the HFD mice (*P* < 0.01 or *P* < 0.05). In contrast, the mRNA expression levels of *GLUT4* and *IRS-1* in SAT and VAT were markedly decreased in the HFD groups compared to those in the STD group (*P* < 0.05). The mRNA expression of *GLUT4* in SAT was significantly increased by RES administration (*P* < 0.01 or *P* < 0.05). Furthermore, the mRNA expression of *GLUT4* and *IRS-1* in VAT was also markedly increased by RES administration (*P* < 0.05).

### Effects of RES treatment on protein expression levels in SAT and VAT in mice

**Figure 7:**
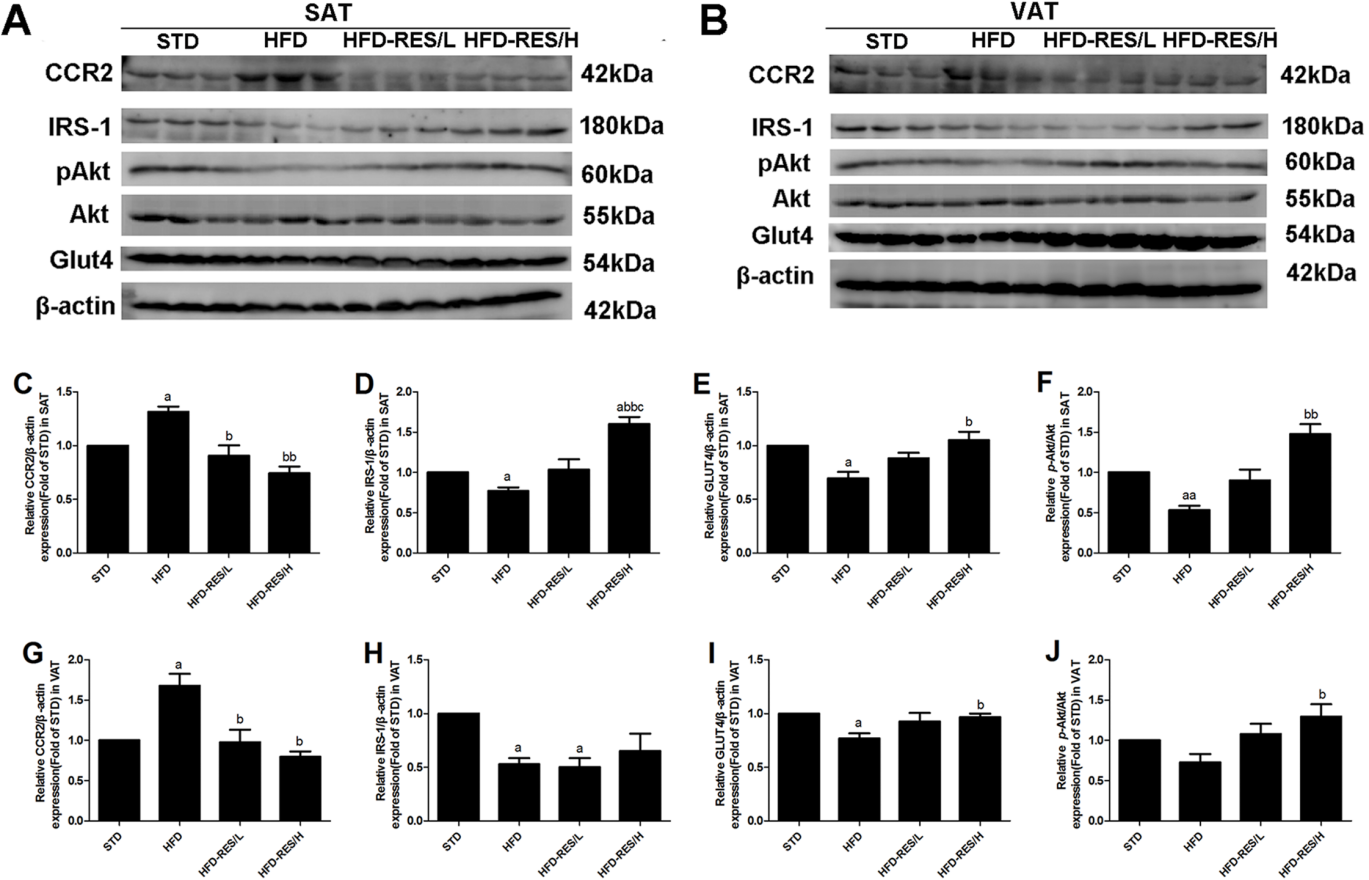
Effects of resveratrol treatment on the protein expression of CCR2, IRS-1, pAkt (phosphorylation at Ser ^473^) and GLUT4 in SAT and VAT. (A) Western blots of CCR2 and insulin signaling molecules in SAT; (B) Western blots of CCR2 and insulin signaling molecules in VAT; (C–F) CCR2, IRS-1, pAkt and GLUT4 protein expression levels in SAT; (G–J) CCR2, IRS-1, pAkt and GLUT4 protein expression levels in VAT. ^a^, *P* < 0.05 and ^aa^, *P* < 0.01 vs. the STD group; ^b^, *P* < 0.05 and ^bb^, *P* < 0.01 vs. the HFD group; ^c^, *P* < 0.05 vs. the HFD-RES/L group. The data are represented as the mean ± SD.

Changes in protein expression of CCR2, GLUT4, IRS-1 and pAkt were measured using Western blot analysis (see Fig. 7). CCR2 protein expression was markedly increased in SAT and VAT in the HFD group compared to that in the STD group (*P* < 0.05). RES administration significantly decreased CCR2 protein expression in SAT and VAT of the HFD groups (*P* < 0.01 or *P* < 0.05). Compared to the STD group, decreased protein expression of IRS-1 and GLUT4 in SAT and VAT in the HFD groups was observed (*P* < 0.05), and RES administration significantly increased expression of IRS-1 and GLUT4 in SAT and VAT (*P* < 0.01 or *P* < 0.05). Furthermore, the protein expression of IRS-1 in the HFD-RES/H group was higher than that in the other three groups (*P* < 0.01 or *P* < 0.05). In addition, the results showed that the protein expression of pAkt in SAT in the HFD group was lower than that in the STD group (*P* < 0.01), and the protein expression of pAkt in SAT in the HFD-RES/H group was higher than that in in the HFD group (*P* < 0.01). Similarly, the protein expression of pAkt in SAT in the HFD-RES/H group was higher than that in the HFD group (*P* < 0.01).

## Discussion

Our study indicates that CCR2 in WAT may play a critical role in macrophage infiltration and the inflammatory response during the development of insulin resistance in HFD-induced obese mice. Our study further demonstrated that RES administration could reverse insulin resistance, decrease CCR2 expression in SAT and VAT, and reduce inflammation and macrophage infiltration of SAT and VAT, thus increasing insulin signaling molecules (such as IRS-1, GLUT4 and pAkt) in HFD-induced obese mice.

Macrophage infiltration into adipose tissues is characteristic of chronic inflammation associated with obesity. Recently, chemokine receptors (such as CCR2) have been proposed to be attractive targets for antiobesity. They interact with their ligands, increasing inflammatory cells in adipose tissue, and further contribute to chronic inflammation and insulin resistance in obesity (Ota, 2013). Increasing evidence has shown that CCR2 could promote the macrophage recruitment and infiltration of tissue in the pathogenesis of insulin resistance (Huh et al., 2017; Kanda et al., 2006). Thus, we studied the role of CCR2 in SAT and VAT during the development of insulin resistance related to obesity. Consistent with the increased expression of serum inflammatory cytokines (such as MCP-1, TNF-α and IL-6) in obese mice, we demonstrated that the expression of CCR2 and macrophage infiltration in SAT and VAT were significantly increased in diet-induced obese mice. This was also observed in other studies of WAT in diet-induced obese rodent models (Kim et al., 2017; Weisberg et al., 2006). In vitro, RES inhibited the mRNA and protein expression in CCR2 of THP-1 monocytes (Cullen et al., 2007). Here, we found that concomitant with a decrease in CCR2 expression and total macrophage F4/80 in SAT and VAT, proinflammatory cytokines in serum and mRNA levels of inflammatory cytokines in WAT were significantly reduced in RES-treated obese mice. These results suggest that CCR2 expression decreased by RES treatment may play a vital role in the reduced migration of total macrophages and subsequent inflammation in adipose tissues.

In population experiments, whether RES administration could improve glucose metabolism disorder is inconclusive. Studies performed in patients with T2DM (Bhatt, Thomas & Nanjan, 2012) and healthy obese men (Timmers et al., 2011) observed the beneficial effects of RES administration for three months and four weeks, respectively, on systemic insulin sensitivity. However, another study conducted in normal-weight, healthy individuals did not demonstrate improved glucose metabolism after RES administration for six weeks (Ghanim et al., 2010). These contradictory results indicate that RES treatment may have a beneficial effect on glucose metabolism disorders in obese individuals rather than normal-weight individuals, and excess adipose tissues may be the target for RES for alleviating disordered glucose mechanisms in individuals. In our study, we observed that RES administration significantly reduced the AUC, improved HOMA-IR and increased insulin sensitivity in HFD-induced obese mice, which suggests that RES improved glucose intolerance and glucose homeostasis. In corroboration with our findings, some rodent studies have also reported that RES treatment ameliorated insulin resistance in HFD animals for eight weeks (Chen et al., 2011; Haohao et al., 2015). Long-term IL-6 or TNF-α treatment inhibits the expression of GLUT4 and IRS-1 in adipose tissue (Hotamisligil et al., 1996; Rotter, Nagaev & Smith, 2003), and in obese patients with T2DM, lower levels of GLUT4 and IRS-1 in VAT were observed (Georgescu et al., 2011; Lancha et al., 2015). In addition, glucose disposal in WAT is affected by misleading insulin signaling via a reduction in the PI3K/pAkt signaling pathway (Muthusamy et al., 2010). The above results indicate that inflammatory cytokines contribute substantially to driving the abnormal insulin signaling cascade of WAT in obesity, leading to a worsening of whole-body insulin resistance and glucose intolerance. Our results demonstrated that long-term RES administration alleviated abnormal insulin signaling cascades in WAT of HFD-induced obese mice by increasing the levels of IRS-1 and GLUT4 and enhancing the phosphorylation level of the Akt protein. In line with our findings, Jimenez-Gomez et al. (2013) also found that chronic administration of RES exerts an anti-inflammatory effect and improves adipose insulin signaling in adipose tissue of high-fat, high-sugar diet-fed rhesus monkeys. Moreover, improved insulin resistance via inhibition of TNF-α and TNF-α-mediated inflammation by RES treatment were observed in 3T3-L1 adipocytes (Zhang, Du & Meng, 2013) and in primary human adipocytes (Chuang et al., 2010). In this paper, we showed that RES administration for 18 weeks markedly decreased the SAT coefficient and prevented dyslipidemia in HFD-fed mice. In agreement with our study, Chen et al. (2017) also reported that RES (400 mg/kg/day) treatment for three months provided protection against diet-induced dyslipidemia in C57BL/6 mice. Studies have demonstrated that RES treatment has fat mobilization and antiobesity effects in rodents (Baile et al., 2011), and also significantly reduced the body weight gain of female Wistar rats and ovariectomized female Wistar rats (Sharma, Sharma & Thungapathra, 2017). In this study, we observed that the RES intervention did not significantly reduce the body weight of mice; however, we detected a trend of reduced body weight in mice treated with RES.

## Conclusion

In conclusion, our results indicate that RES administration could increase CCR2 expression in WAT, alleviate inflammation and macrophage infiltration to improve the expression of classical markers of the insulin signaling cascade in SAT and VAT, and maintain glucose metabolic homeostasis in diet-induced obese mice. Thus, the current study highlights the potential clinical utility of RES administration in attenuating macrophage-induced inflammation in adipose tissue and preventing obesity-related metabolic diseases.

## Supplemental Information

10.7717/peerj.5173/supp-1 Supplemental Information 1 Raw data.

10.7717/peerj.5173/supp-2 Supplemental Information 2 Raw pictures for gel and blots.

10.7717/peerj.5173/supp-3 Supplemental Information 3 The mRNA raw data.

## Additional Information and Declarations

### Field Study Permissions

The following information was supplied relating to field study approvals (i.e., approving body and any reference numbers): Field experiments were approved by the Xinxiang Medical University Animal Care and Use Committee (XXMU-2016-0007).

### Data Availability

The following information was supplied regarding data availability: The raw data are provided in the Supplemental Files.

## References

[ref-1] Baile CA, Yang JY, Rayalam S, Hartzell DL, Lai CY, Andersen C, Della-Fera MA (2011). Effect of resveratrol on fat mobilization. Annals of the New York Academy of Sciences.

[ref-2] Bhatt JK, Thomas S, Nanjan MJ (2012). Resveratrol supplementation improves glycemic control in type 2 diabetes mellitus. Nutrition Research.

[ref-3] Bolus WR, Gutierrez DA, Kennedy AJ, Anderson-Baucum EK, Hasty AH (2015). CCR2 deficiency leads to increased eosinophils, alternative macrophage activation, and type 2 cytokine expression in adipose tissue. Journal of Leukocyte Biology.

[ref-4] Chen L, Wang T, Chen G, Wang N, Gui L, Dai F, Fang Z, Zhang Q, Lu Y (2017). Influence of resveratrol on endoplasmic reticulum stress and expression of adipokines in adipose tissues/adipocytes induced by high-calorie diet or palmitic acid. Endocrine.

[ref-5] Chen LL, Zhang HH, Zheng J, Hu X, Kong W, Hu D, Wang SX, Zhang P (2011). Resveratrol attenuates high-fat diet-induced insulin resistance by influencing skeletal muscle lipid transport and subsarcolemmal mitochondrial β-oxidation. Metabolism.

[ref-6] Chuang CC, Martinez K, Xie G, Kennedy A, Bumrungpert A, Overman A, Jia W, McIntosh MK (2010). Quercetin is equally or more effective than resveratrol in attenuating tumor necrosis factor-α–mediated inflammation and insulin resistance in primary human adipocytes. American Journal of Clinical Nutrition.

[ref-7] Cullen JP, Morrow D, Jin Y, von Offenberg Sweeney N, Sitzmann JV, Cahill PA, Redmond EM (2007). Resveratrol inhibits expression and binding activity of the monocyte chemotactic protein-1 receptor, CCR2, on THP-1 monocytes. Atherosclerosis.

[ref-8] Côté CD, Rasmussen BA, Duca FA, Zadeh-Tahmasebi M, Baur JA, Daljeet M, Breen DM, Filippi BM, Lam TKT (2015). Resveratrol activates duodenal Sirt1 to reverse insulin resistance in rats through a neuronal network. Natural Medicines.

[ref-9] Ding S, Fan Y, Zhao N, Yang H, Ye X, He D, Jin X, Liu J, Tian C, Li H, Xu S, Ying C (2014). High-fat diet aggravates glucose homeostasis disorder caused by chronic exposure to bisphenol A. Journal of Endocrinology.

[ref-10] Ding S, Jiang J, Zhang G, Bu Y, Zhang G, Zhao X (2017). Resveratrol and caloric restriction prevent hepatic steatosis by regulating SIRT1-autophagy pathway and alleviating endoplasmic reticulum stress in high-fat diet-fed rats. PLOS ONE.

[ref-11] Fjeldborg K, Pedersen SB, Moller HJ, Christiansen T, Bennetzen M, Richelsen B (2014). Human adipose tissue macrophages are enhanced but changed to an anti-inflammatory profile in obesity. Journal of Immunology Research.

[ref-12] Georgescu A, Popov D, Constantin A, Nemecz M, Alexandru N, Cochior D, Tudor A (2011). Dysfunction of human subcutaneous fat arterioles in obesity alone or obesity associated with Type 2 diabetes. Clinical Science.

[ref-13] Ghanim H, Sia CL, Abuaysheh S, Korzeniewski K, Patnaik P, Marumganti A, Chaudhuri A, Dandona P (2010). An antiinflammatory and reactive oxygen species suppressive effects of an extract of Polygonum cuspidatum containing resveratrol. Journal of Clinical Endocrinology and Metabolism.

[ref-14] Haohao Z, Guijun Q, Juan Z, Wen K, Lulu C (2015). Resveratrol improves high-fat diet induced insulin resistance by rebalancing subsarcolemmal mitochondrial oxidation and antioxidantion. Journal of Physiology and Biochemistry.

[ref-15] Harford KA, Reynolds CM, McGillicuddy FC, Roche HM (2011). Fats, inflammation and insulin resistance: insights to the role of macrophage and T-cell accumulation in adipose tissue. Proceedings of the Nutrition Society.

[ref-16] Hotamisligil GS, Peraldi P, Budavari A, Ellis R, White MF, Spiegelman BM (1996). IRS-1-mediated inhibition of insulin receptor tyrosine kinase activity in TNF-α-and obesity-induced insulin resistance. Science.

[ref-17] Huh JH, Kim HM, Lee ES, Kwon MH, Lee BR, Ko HJ, Chung CH (2017). Dual CCR2/5 antagonist attenuates obesity-induced insulin resistance by regulating macrophage recruitment and M1/M2 status. Obesity.

[ref-18] Jimenez-Gomez Y, Mattison JA, Pearson KJ, Martin-Montalvo A, Palacios HH, Sossong AM, Ward TM, Younts CM, Lewis K, Allard JS, Longo DL, Belman JP, Malagon MM, Navas P, Sanghvi M, Moaddel R, Tilmont EM, Herbert RL, Morrell CH, Egan JM, Baur JA, Ferrucci L, Bogan JS, Bernier M, de Cabo R (2013). Resveratrol improves adipose insulin signaling and reduces the inflammatory response in adipose tissue of rhesus monkeys on high-fat, high-sugar diet. Cell Metabolism.

[ref-19] Kanda H, Tateya S, Tamori Y, Kotani K, Hiasa K, Kitazawa R, Kitazawa S, Miyachi H, Maeda S, Egashira K, Kasuga M (2006). MCP-1 contributes to macrophage infiltration into adipose tissue, insulin resistance, and hepatic steatosis in obesity. Journal of Clinical Investigation.

[ref-20] Kelly T, Yang W, Chen CS, Reynolds K, He J (2008). Global burden of obesity in 2005 and projections to 2030. International Journal of Obesity.

[ref-21] Kim J, Chung K, Choi C, Beloor J, Ullah I, Kim N, Lee KY, Lee SK, Kumar P (2016). Silencing CCR2 in macrophages alleviates adipose tissue inflammation and the associated metabolic syndrome in dietary obese mice. Molecular Therapy—Nucleic Acids.

[ref-22] Kim HM, Kim YM, Huh JH, Lee ES, Kwon MH, Lee BR, Ko HJ, Chung CH (2017). α-Mangostin ameliorates hepatic steatosis and insulin resistance by inhibition C-C chemokine receptor 2. PLOS ONE.

[ref-23] Kim HM, Lee ES, Lee BR, Yadav D, Kim YM, Ko HJ, Park KS, Lee EY, Chung CH (2015). C-C chemokine receptor 2 inhibitor ameliorates hepatic steatosis by improving ER stress and inflammation in a type 2 diabetic mouse model. PLOS ONE.

[ref-24] Lagouge M, Argmann C, Gerhart-Hines Z, Meziane H, Lerin C, Daussin F, Messadeq N, Milne J, Lambert P, Elliott P, Geny B, Laakso M, Puigserver P, Auwerx J (2006). Resveratrol improves mitochondrial function and protects against metabolic disease by activating SIRT1 and PGC-1α. Cell.

[ref-25] Lancha A, Lopez-Garrido S, Rodriguez A, Catalan V, Ramirez B, Valenti V, Moncada R, Silva C, Gil MJ, Salvador J, Fruhbeck G, Gomez-Ambrosi J (2015). Expression of syntaxin 8 in visceral adipose tissue is increased in obese patients with type 2 diabetes and related to markers of insulin resistance and inflammation. Archives of Medical Research.

[ref-26] Marmugi A, Ducheix S, Lasserre F, Polizzi A, Paris A, Priymenko N, Bertrand-Michel J, Pineau T, Guillou H, Martin PGP, Mselli-Lakhal L (2012). Low doses of bisphenol A induce gene expression related to lipid synthesis and trigger triglyceride accumulation in adult mouse liver. Hepatology.

[ref-27] Michaud A, Drolet R, Noel S, Paris G, Tchernof A (2012). Visceral fat accumulation is an indicator of adipose tissue macrophage infiltration in women. Metabolism.

[ref-28] Muthusamy VS, Saravanababu C, Ramanathan M, Bharathi Raja R, Sudhagar S, Anand S, Lakshmi BS (2010). Inhibition of protein tyrosine phosphatase 1B and regulation of insulin signalling markers by caffeoyl derivatives of chicory (Cichorium intybus) salad leaves. British Journal of Nutrition.

[ref-29] Ota T (2013). Chemokine systems link obesity to insulin resistance. Diabetes & Metabolism Journal.

[ref-30] Pan QR, Ren YL, Liu WX, Hu YJ, Zheng JS, Xu Y, Wang G (2015). Resveratrol prevents hepatic steatosis and endoplasmic reticulum stress and regulates the expression of genes involved in lipid metabolism, insulin resistance, and inflammation in rats. Nutrition Research.

[ref-31] Park S, Park HL, Lee SY, Nam JH (2016). Characteristics of adipose tissue macrophages and macrophage-derived insulin-like growth factor-1 in virus-induced obesity. International Journal of Obesity.

[ref-32] Pradhan A (2007). Obesity, metabolic syndrome, and type 2 diabetes: inflammatory basis of glucose metabolic disorders. Nutrition Reviews.

[ref-33] Qatanani M, Lazar MA (2007). Mechanisms of obesity-associated insulin resistance: many choices on the menu. Genes & Development.

[ref-34] Rotter V, Nagaev I, Smith U (2003). Interleukin-6 (IL-6) induces insulin resistance in 3T3-L1 adipocytes and is, like IL-8 and tumor necrosis factor-α, overexpressed in human fat cells from insulin-resistant subjects. Journal of Biological Chemistry.

[ref-35] Schueller K, Pignitter M, Somoza V (2015). Sulfated and glucuronated trans-resveratrol metabolites regulate chemokines and sirtuin-1 expression in U-937 macrophages. Journal of Agricultural and Food Chemistry.

[ref-36] Sharma R, Sharma NK, Thungapathra M (2017). Resveratrol regulates body weight in healthy and ovariectomized rats. Nutrition and Metabolism.

[ref-37] Sullivan TJ, Miao Z, Zhao BN, Ertl LS, Wang Y, Krasinski A, Walters MJ, Powers JP, Dairaghi DJ, Baumgart T, Seitz LC, Berahovich RD, Schall TJ, Jaen JC (2013). Experimental evidence for the use of CCR2 antagonists in the treatment of type 2 diabetes. Metabolism.

[ref-38] Timmers S, Konings E, Bilet L, Houtkooper RH, van de Weijer T, Goossens GH, Hoeks J, van der Krieken S, Ryu D, Kersten S, Moonen-Kornips E, Hesselink MKC, Kunz I, Schrauwen-Hinderling VB, Blaak E, Auwerx J, Schrauwen P (2011). Calorie restriction-like effects of 30 days of resveratrol supplementation on energy metabolism and metabolic profile in obese humans. Cell Metabolism.

[ref-39] Wang M, Gao XJ, Zhao WW, Zhao WJ, Jiang CH, Huang F, Kou JP, Liu BL, Liu K (2013). Opposite effects of genistein on the regulation of insulin-mediated glucose homeostasis in adipose tissue. British Journal of Pharmacology.

[ref-40] Weisberg SP, Hunter D, Huber R, Lemieux J, Slaymaker S, Vaddi K, Charo I, Leibel RL, Ferrante AW (2006). CCR2 modulates inflammatory and metabolic effects of high-fat feeding. Journal of Clinical Investigation.

[ref-41] Xu H, Barnes GT, Yang Q, Tan G, Yang D, Chou CJ, Sole J, Nichols A, Ross JS, Tartaglia LA, Chen H (2003). Chronic inflammation in fat plays a crucial role in the development of obesity-related insulin resistance. Journal of Clinical Investigation.

[ref-42] Zhang HY, Du ZX, Meng X (2013). Resveratrol prevents TNFα-induced suppression of adiponectin expression via PPARγ activation in 3T3-L1 adipocytes. Clinical and Experimental Medicine.

[ref-43] Zhao W, Li A, Feng X, Hou T, Liu K, Liu B, Zhang N (2016). Metformin and resveratrol ameliorate muscle insulin resistance through preventing lipolysis and inflammation in hypoxic adipose tissue. Cellular Signalling.

[ref-44] Zheng C, Yang Q, Xu C, Shou P, Cao J, Jiang M, Chen Q, Cao G, Han Y, Li F, Cao W, Zhang L, Shi Y, Wang Y (2015). CD11b regulates obesity-induced insulin resistance via limiting alternative activation and proliferation of adipose tissue macrophages. Proceedings of the National Academy of Sciences of the United States of America.

