# Supplementary material for: Resveratrol reduces the inflammatory response in adipose tissue and improves adipose insulin signaling in high-fat diet-fed mice
Source: PeerJ. 2018 Jun 29;6:e5173. doi: 10.7717/peerj.5173 (PMC6027658; doi:10.7717/peerj.5173)
Supplement: Supplemental Information 1 [file peerj-06-5173-s001.pdf]

Table 1. The body weight curve in four groups (g, Mean)

Week/Body weight	STD	HFD	HFD-RES/L	HFD-RES/H
0	21.95	22.30	22.09	21.89
3	25.86	27.19	26.49	26.05
6	27.75	31.04	32.28	31.01
9	28.17	32.68	34.24	35.16
12	29.74	34.68	36.05	37.65
15	31.83	37.07	39.00	38.95
18	32.62	42.24	40.52	39.28

Body weight(0 week)

STD	HFD	HFD-RES/L	HFD-RES/H
22.59	23.3	20.9	20.4
23.79	23.4	22.5	22.3
20.49	21.0	22.3	21.2
21.29	24.0	22.4	21.0
21.59	21.8	23.8	23.1
23.29	23.0	22.6	20.8
21.69	22.5	21.2	22.4
21.49	21.5	21.3	23.6
22.49	21.0	21.2	21.5
20.79	21.5	22.7	22.6

Body weight(3 week)

STD	HFD	HFD-RES/L	HFD-RES/H
28.2	26.1	25.9	25.4
26.5	25.8	27.5	26.8
23.6	25.6	26.6	28.1
22.7	29.0	26.9	25.7
27.8	28.6	27.0	24.5
28.5	27.3	27.5	26.2
24.9	27.0	26.7	25.7
24.5	26.4	25.6	25.5
26.9	27.8	25.0	26.7
25.0	28.3	26.2	25.9

Body weight(6 week)

STD	HFD	HFD-RES/L	HFD-RES/H
31.0	30.8	32.1	30.4
29.8	29.3	32.6	32.6
27.2	29.4	32.5	32.1
26.9	31.5	30.4	29.3

31.4	31.6	30.6	29.8
28.3	33.3	32.7	31.9
26.4	31.6	33.2	31.4
26.5	31.1	30.1	30.7
31.2	30.7	34.2	30.3
28.8	31.1	34.4	31.6

Body weight(9 week)

STD	HFD	HFD-RES/L	HFD-RES/H
29.4	32.4	36.1	37.6
26.6	30.8	36.8	35.8
24.8	29.3	36.0	36.0
23.7	33.4	34.7	33.5
28.8	34.9	35.5	36.9
25.0	34.0	36.9	34.2
25.8	35.2	30.3	33.1
30.4	32.7	31.2	34.4
27.0	33.1	31.7	34.9
28.2	31.0	33.2	35.2

Body weight(12 week)

STD	HFD	HFD-RES/L	HFD-RES/H
32.1	36.0	38.6	37.9
28.0	31.3	37.6	37.4
27.6	32.3	37.1	38.1
26.9	35.6	36.2	38.8
32.0	35.0	35.5	39.8
33.4	35.4	37.9	38.4
28.3	36.9	32.8	37.0
27.6	35.7	33.3	35.2
30.5	34.2	36.4	36.2
31.0	34.4	35.1	37.7

Body weight(15 week)

STD	HFD	HFD-RES/L	HFD-RES/H
31.8	36.8	39.3	37.6
32.8	32.4	41.2	38.5
29.0	35.7	40.5	39.7
30.1	39.0	41.9	42.0
32.9	38.1	39.0	41.1
30.2	37.9	41.5	40.2
31.4	38.9	33.4	38.9
34.3	39.0	35.8	37.3
33.3	36.6	39.0	37.0
32.5	36.3	38.4	37.2

Body weight(18 week)

STD	HFD	HFD-RES/L	HFD-RES/H
34.3	40.9	41.1	38.3
32.8	42.5	40.7	39.2
31.1	43.4	41.2	38.5
29.3	42.9	42.1	41.4
31.3	41.1	41.7	41.8
30.5	42.7	41.5	39.6
34.5	43.5	36.4	38.2
34.6	42.9	38.3	37.0
35.3	42.2	41.9	38.7
32.5	40.3	40.3	40.1

Table 2. The body weight gain in four groups (g)

STD	HFD	HFD-RES/L	HFD-RES/H
10.71	17.6	20.2	17.9
11.71	19.1	18.2	16.9
9.01	22.4	18.9	17.3
10.61	18.9	19.7	20.4
8.01	19.3	17.9	18.7
9.71	19.7	18.9	18.8
7.21	21.0	15.2	15.8
12.81	21.4	17.0	13.4
13.11	21.2	20.7	17.2
12.81	18.8	17.6	17.5

Table 3. The SAT coefficient and VAT coefficient in four groups (Mean±SD)

	STD	HFD	HFD-RES/L	HFD-RES/H
SAT coefficient(%)	1.66±0.28	2.84±0.29	2.51±0.25	2.49±0.17
VAT coefficient(%)	0.80±0.18	1.46±0.15	1.51±0.20	1.48±0.12

SAT coefficient(%)

STD	HFD	HFD-RES/L	HFD-RES/H
1.31	3.03	2.63	2.35
1.62	2.66	2.31	2.27
1.19	2.56	2.60	2.52
1.71	2.38	2.73	2.75
2.11	2.65	2.59	2.61
2.01	2.93	2.31	2.68
1.65	3.08	2.55	2.59
1.50	3.24	2.38	2.49

1.67	2.70	2.65	2.48
1.85	3.15	2.38	2.24

VAT coefficient(%)

STD	HFD	HFD-RES/L	HFD-RES/H
0.87	1.66	1.34	1.489
1.10	1.32	1.20	1.68
0.68	1.34	1.46	1.58
0.75	1.40	1.69	1.47
1.02	1.36	1.53	1.32
0.66	1.52	1.64	1.59
0.87	1.36	1.79	1.28
0.52	1.33	1.67	1.49
0.68	1.56	1.55	1.40
0.83	1.71	1.24	1.47

Table 4. The glucose in different times in IPGTT (mmol/L, Mean)

Time(Min)	STD	HFD	HFD-RES/L	HFD-RES/H
0	6.32	10.14	7.08	6.96
15	22.32	28.70	24.62	24.22
30	25.00	30.54	27.08	26.52
60	13.92	23.02	22.36	21.42
120	8.40	14.50	14.12	13.60

Glucose(mmol/L)

group	0 min	15 min	30 min	60 min	120 min	AUC
STD	5.9	21.3	24.7	13.7	7.2	1752.0
STD	6.6	23.3	24.5	12.8	8.1	1769.25
STD	7.1	22.6	25.9	14.9	8.8	1909.5
STD	6.2	21.5	24.8	15.2	9.3	1890.0
STD	5.8	22.9	25.1	13.0	8.6	1794.75
HFD	8.8	29.1	29.9	20.3	14.6	2526.75
HFD	10.2	28.6	30.1	23.6	15.4	2706.75
HFD	11.7	27.5	31.2	24.3	13.4	2697.75
HFD	9.6	28.5	29.7	22.5	16.0	2660.25
HFD	10.4	29.8	31.8	24.4	13.1	2731.5
HFD-RES/L	7.0	29.1	31.1	20.5	15.8	2585.25
HFD-RES/L	6.6	27.6	29.6	24.6	16.3	2725.5
HFD-RES/L	7.7	26.5	28.4	25.1	14.2	2649.75
HFD-RES/L	6.2	27.1	29.6	25.2	13.9	2670.0
HFD-RES/L	7.9	26.8	28.7	21.4	13.3	2469.0
HFD-RES/H	7.2	28.2	30.3	23.5	15.5	2681.25
HFD-RES/H	6.5	27.9	29.9	24.0	16.4	2712.0
HFD-RES/H	7.4	25.3	27.5	22.3	15.1	2510.25

HFD-RES/H	6.6	26.6	28.5	25.7	14.7	2687.25
HFD-RES/H	7.1	27.1	28.4	21.6	13.8	2484.75

Table 5. The AUC, HOMA-IR and Insulin sensitivity index in IPGTT (Mean±SD)

	STD	HFD	HFD-RES/L	HFD-RES/H
AUC(mmol/L per min)	1823.1±71.94	2664.6±81.20	2461.5±70.43	2384.1±110.60
HOMA-IR	0.59±0.06	0.83±0.04	0.77±0.04	0.75±0.05
Insulin sensitivity index	0.39±0.015	0.34±0.006	0.35±0.007	0.36±0.010

Table 6. The serum lipid in four groups (Mean±SD)

mmol/L	STD	HFD	HFD-RES/L	HFD-RES/H
Serum TG	0.69±0.09	1.40±0.10	1.23±0.09	1.21±0.08
Serum TC	2.08±0.24	3.75±0.15	3.51±0.15	3.44±0.29
Serum HDL	1.86±0.11	1.33±0.09	1.49±0.12	1.55±0.13
Serum LDL	0.36±0.06	0.74±0.09	0.64±0.06	0.63±0.05

Table 7. The serum inflammation markers in four groups (Mean±SD)

(pg/mL)	STD	HFD	HFD-RES/L	HFD-RES/H
MCP-1	552.11±35.54	754.70±47.01	702.97±32.18	694.60±39.85
TNF-α	353.04±26.14	455.48±35.48	414.74±34.20	394.52±30.11
IL-6	510.09±58.55	814.12±62.93	735.81±48.74	722.15±53.81

Table 8. The F4/80 of VAT and SAT (relative to STD group) in four groups (Mean±SD)

	STD	HFD	HFD-RES/L	HFD-RES/H
VAT	1	1.84±0.21	1.56±0.15	1.37±0.11
SAT	1	2.74±0.26	2.22±0.17	2.14±0.16

Table 9. The mRNA expressions of VAT (relative to STD group) in four groups (Mean±SD)

	STD	HFD	HFD-RES/L	HFD-RES/H
CCR2	1	1.72±0.16	1.49±0.14	1.40±0.15
MCP-1	1	4.38±0.61	2.81±0.31	2.65±0.32
TNF-α	1	1.98±0.31	1.69±0.15	1.45±0.15
IL-6	1	3.89±0.35	3.49±0.22	3.40±0.19
GLUT4	1	0.65±0.14	1.01±0.17	1.20±0.16
IRS-1	1	0.67±0.13	0.87±0.12	0.96±0.14

Table 10. The mRNA expressions of SAT (relative to STD group) in four groups (Mean±SD)

	STD	HFD	HFD-RES/L	HFD-RES/H

CCR2	1	1.35±0.15	0.97±0.19	0.75±0.13
MCP-1	1	2.93±0.27	2.82±0.25	2.51±0.20
TNF-α	1	2.16±0.26	1.98±0.24	1.62±0.14
IL-6	1	3.14±0.33	2.90±0.31	2.60±0.19
GLUT4	1	0.72±0.13	1.10±0.18	1.20±0.22
IRS-1	1	0.59±0.16	0.79±0.23	0.99±0.19

Table 11. The protein expressions of VAT in four groups (Mean±SD)

	STD	HFD	HFD-RES/L	HFD-RES/H
CCR2	1	1.315±0.049	0.907±0.096	0.743±0.062
pAKT	1	0.532±0.053	0.902±0.134	1.477±0.123
GLUT4	1	0.697±0.059	0.882±0.051	1.051±0.078
IRS-1	1	0.769±0.047	1.036±0.126	1.603±0.086

Table 12. The protein expressions of SAT in four groups (Mean±SD)

	STD	HFD	HFD-RES/L	HFD-RES/H
CCR2	1	1.677±0.148	0.978±0.157	0.797±0.069
pAKT	1	0.732±0.100	1.080±0.127	1.300±0.154
GLUT4	1	0.771±0.044	0.927±0.079	0.968±0.032
IRS-1	1	0.530±0.056	0.501±0.084	0.653±0.161
